# *TCF7L2* as a target of peripheral artery disease in patients with type 2 diabetes: A 2-sample Mendelian randomization and bioinformatics study

**DOI:** 10.1097/MD.0000000000041431

**Published:** 2025-02-14

**Authors:** Jie Liu, XingDe Liu, Rui Rao, Wen Li

**Affiliations:** a Department of Basic Medical College, Guizhou University of Traditional Chinese Medicine, Guiyang, Guizhou, China; b Department of Cardiology, Longli Hospital of Traditional Chinese Medicine, Qiannan, Guizhou, China; c Department of Cardiology, Second Affiliated Hospital of Guizhou University of Traditional Chinese Medicine, Guiyang, Guizhou, China; d Department of Endocrinology, Second Affiliated Hospital of Guizhou University of Traditional Chinese Medicine, Guiyang, Guizhou, China.

**Keywords:** bioinformatics, Mendelian randomization, peripheral artery disease, *TCF7L2*, type 2 diabetes

## Abstract

This study examines the causal relationship between type 2 diabetes (T2D) and peripheral artery disease (PAD) and their potential mechanisms based on the analysis of the Gene Expression Omnibus database and 2-sample Mendelian randomization (MR). The first part involved a 2-sample MR study and a comprehensive meta-analysis. Differences in the results were assessed using inverse-variance weighting. Heterogeneity was examined using the Cochrane *Q* statistical test. The leave-one-out method was applied for sensitivity analysis. The potential horizontal pleiotropic effect was assessed using the MR-Egger intercept technique. The second part involved differential gene analysis and weighted gene coexpression network analysis. Subsequently, we overlapped and consolidated the results from the 2 parts to identify the key genes between them. MR analysis results suggested a statistically significant correlation between the incidence of PAD and T2D (odds ratio: 1.22, 95% confidence interval: 1.13–1.32, *P* = 3.74e−07). We anticipated a pivotal role for *TCF7L2* in PAD and T2D. T2D was significantly associated with PAD risk. Simultaneously, the study deepened our understanding of the underlying mechanisms of both diseases, proposing *TCF7L2* as a promising target.

## Graphical abstract

**Figure:**
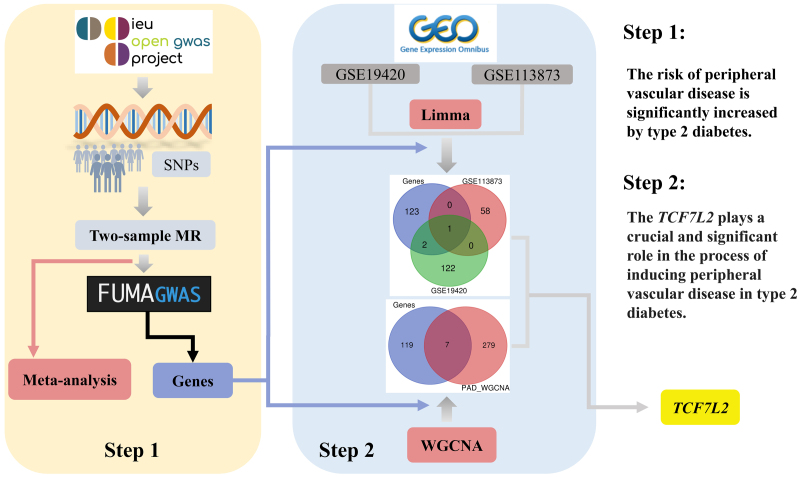

## 1. Introduction

Type 2 diabetes (T2D) presents a significant global health risk, resulting in an increased socioeconomic burden.^[1]^ Between 1980 and 2019, the global prevalence of adult diabetes surged from 108 to 463 million cases.^[2]^ By 2030, the global incidence rate of T2D will reach 7079 cases per 100,000 individuals, based on epidemiological data from the global burden of disease. T2D is responsible for over 1 million deaths annually, ranking as the 9th leading cause of mortality.^[3]^ Between 1990 and 2019, the global incidence and disability-adjusted life years of T2D in young- and middle-aged individuals (aged 15–39 years) exhibited a consistent upward trend.^[4]^ T2D markedly increases the risk of vascular diseases and affects both small and large blood vessels.^[5]^ One report has revealed a steady increase in the global number of patients with peripheral artery disease (PAD),^[6]^ especially among the elderly, and a higher incidence rate in females than in males.^[7]^ PAD associated with T2D progressively reduces quality of life and increases the socioeconomic burden.^[8]^ Therefore, the prevention and early management of PAD receive significant societal attention.^[9]^ T2D is estimated to be the main factor contributing to PAD.^[10]^ Elevated fasting blood glucose level is currently a significant risk factor for the global age-standardized death rate linked to PAD.^[11]^ Early intervention in PAD and strict control of the associated risks can effectively prevent cardiovascular events and mitigate adverse limb events.^[12]^ An increasing body of evidence shows a strong association between T2D and PAD, particularly in the field of genetics.^[13]^ Unfortunately, there is insufficient understanding and insight into peripheral arterial disease among both the high-risk population and healthcare professionals.^[14]^

Mendelian randomization (MR) is frequently employed in genetic epidemiology for causal inference.^[15]^ Genetic variations, typically in the form of single-nucleotide polymorphisms (SNPs), act as unbiased markers of modifiable risk factors.^[16]^ This aids in exploring the causal impact of exposure on the outcomes.^[17]^ The MR technique employs instrumental variables (IVs) to systematically investigate the impact of genetic variation,^[18]^ this assesses the potential existence of a causal relationship between exposure factors and outcomes.^[19]^

Therefore, we aimed to investigate the causal relationship and potential links between T2D and PAD using the Gene Expression Omnibus (GEO) database and 2-sample MR analysis, thereby enhancing our understanding of these diseases and offering insights for future investigations.

## 2. Methods

### 2.1. Study design

**Figure 1.**
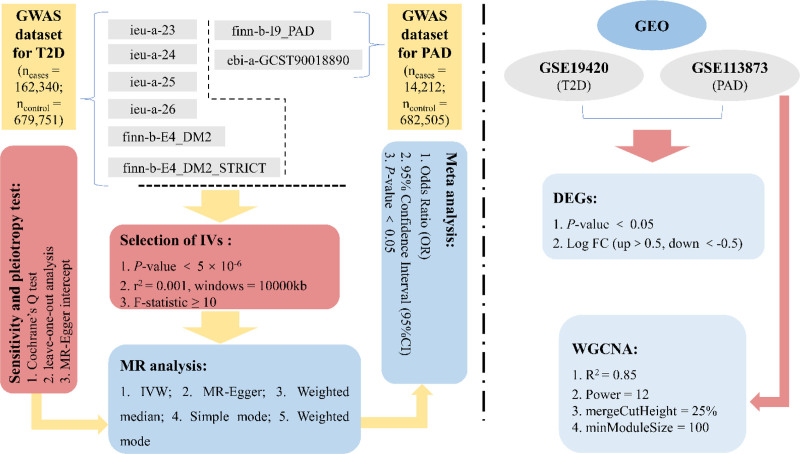
Study design flow chart. DEGs = differentially expressed genes, FC = fold change, GEO = Gene Expression Omnibus, GWAS = genome-wide association studies, IVs = instrumental variables, IVW = inverse variance weighting, MR = Mendelian randomization, PAD = peripheral artery disease, T2D = type 2 diabetes, WGCNA = weighted gene coexpression network analysis.

An overview of the experimental design is shown in Figure 1. In the initial phase, we conducted a 2-sample MR study using the genome-wide association studies (GWAS) database. This study aimed to systematically evaluate the potential causative association between T2D and PAD. In the second phase, we analyzed the GEO database to investigate the relationship between T2D and PAD. Through differentially expressed genes (DEGs) and weighted gene coexpression network analysis (WGCNA) analyses, we identified the corresponding core genes that were utilized in subsequent research. This study was reported following the guidelines of Strengthening the Reporting of Observational Studies in Epidemiology Using Mendelian Randomization statement.^[20]^

### 2.2. Data source and instrument selection

In the initial phase, we performed a 2-sample MR using data from GWAS (https://gwas.mrcieu.ac.uk/) that we obtained independently from the DIAGRAM, FinnGen, and PubMed datasets. This analysis included 6 datasets associated with T2D and 2 datasets associated with PAD. The aggregated T2D data consisted of 4 datasets from the DIAGRAM database^[21-23]^ and 2 datasets from the FinnGen database. The aggregated PAD data included 1 dataset from the FinnGen database and another from PubMed.^[24]^ To alleviate the population stratification bias, this study specifically chose data from individuals of European ancestry.^[25]^ IVs had to meet the following dual criteria concurrently,^[26]^ SNPs with *P* = 5e−06; linkage disequilibrium clustering of SNPs within an *r*^2^ = 0.001 and window = 10,000 kb. The *F*-statistic was used to detect weak IVs. The *F*-statistic ≥ 10 was considered sufficient to mitigate bias caused by weak instruments.^[27]^ The second phase included T2D data retrieved from the GSE19420 dataset and PAD data sourced from the GSE113873 dataset. Data were obtained from the GEO database (https://www.ncbi.nlm.nih.gov/geo/). Both datasets employed the “expression profiling by array” methodology and utilized skeletal muscle samples.

### 2.3. MR analysis

In each cohort, we excluded SNPs with a *P* > 5e−06,^[28]^ which were considered significant variants associated with the phenotype, for subsequent analysis. We set a criterion to exclude linkage disequilibrium, with an *r*^2^ threshold of 0.001 and a maximum distance threshold of 10,000 kb.^[29]^ SNP with *F*-statistic ≥ 10 was evaluated as strong IVs.^[30]^ The *F*-statistic is determined by the genetic variance (*R*^2^), sample size (*N*), and the number of IVs (*κ*). It is calculated using the formula: *F* = *R*^2^ (*N* − *κ* − 1)/*κ* (1 − *R*^2^)^31]^ *R*^2^ represents the proportion of variance explained by the genetic instruments and is defined as: *R*^2^ = 2 EAF (1 − EAF) *β*^2^, where EAF is the effect allele frequency, and 𝛽 denotes the estimated genetic effect on the exposure.^[32]^ We searched the identified SNPs in PhenoScanner (www.phenoscanner.medschl.cam.ac.uk) to exclude potential confounders.^[33]^ We used the “Two Sample MR” package in R software (version 4.3.2) for MR analyses^[34]^ and applied the inverse-variance weighting (IVW)^[35]^ main methods to evaluate the relationship between T2D and PAD. By employing the leave-one-out method^[36]^ and simultaneously calculating the Cochrane *Q* statistical test,^[35]^ we assessed the heterogeneity and sensitivity of the SNPs. We employed the MR-Egger intercept method to assess potential horizontal pleiotropy.^[37]^ The retained IVs underwent gene annotation using the FUMAGWAS database (https://fuma.ctglab.nl/), priming them for subsequent analyses.

### 2.4. Meta-analysis

We used the “metafor” package in R software for meta-analyses.^[38]^ The “Two Sample MR” package was utilized to odds ratio (OR), 95% confidence interval (CI), and *P* values for individual T2D datasets in comparison to the PAD dataset. Following that, we utilized a fixed-effects model for both overall and subgroup effect analyses.

### 2.5. Differentially expressed genes

We used the “limma” package in R software for DEG processing.^[39]^ Initially, we scrutinized the raw data, excluded unnecessary information, and concisely preserved only the essential data representing both the control and case groups. Ultimately, both GSE19420 (*N*_genes_ = 23,322) and GSE113873 (*N*_genes_ = 19,916) contained 22 samples. We computed the fold change (FC), which represents the ratio of gene expression levels between the 2 conditions (including both the treatment and control groups), and determined the associated *P* values. Genes with *P* < .05 and log FC > 0.5 were designated as upregulated, whereas those with *P* < .05 and log FC < −0.5 were classified as downregulated. We generated volcano plots for DEGs with the “ggVolcano” package.

### 2.6. Weighted gene coexpression network analysis

The “WGCNA” package in the R software was used for bioinformatics analysis.^[40]^ We conducted a clustering analysis using 22 samples and 19,916 genes retained from GSE113873. Initially, the presence of outliers in the dataset was assessed. We selected a soft power threshold of 12 (scale-free *R*^2^ = 0.85). The adjacency matrix was methodologically converted into a topological overlap matrix (TOM). The TOM quantitatively describes the node similarity through weighted correlation comparisons between pairs of nodes and other nodes.^[41]^ Subsequently, we established filtering criteria to identify stratified clustering modules: each module had to consist of at least 100 genes (minModuleSize = 100); merged modules with similarity through hierarchical clustering (mergeCutHeight = 0.25); and the Pearson correlation coefficient was employed to gauge the linear association between pairs of genes, thereby assessing their similarity in expression patterns. Finally, the module was identified with *P* < .01, and the highest absolute score within the case group was identified as the core module associated with the case phenotype. The genes in the core module were extracted for further analysis.

### 2.7. Statistical analysis

The primary methodology utilized in our study to evaluate the causal correlation between T2D and PAD was the IVW method. To determine acceptance of the null hypothesis, we set the significance level at *P* < .05. To assess the heterogeneity and sensitivity of the IVs, we employed the leave-one-out method in conjunction with the Cochran *Q* statistical test. The leave-one-out method enabled direct observation of sensitivity among IVs. Heterogeneity was considered present when the *P* value from the *Q* test was <.05. To enhance the robustness of the MR results, we applied the MR-Egger intercept method to assess potential horizontal pleiotropy effects. A significance level below *P* < .05 indicated the presence of pleiotropic effects. In other statistical tests conducted in the present study, *P* < .05 indicated acceptance of the null hypothesis.

## 3. Results

### 3.1. MR analysis results of T2D to PAD

**Figure 2.**
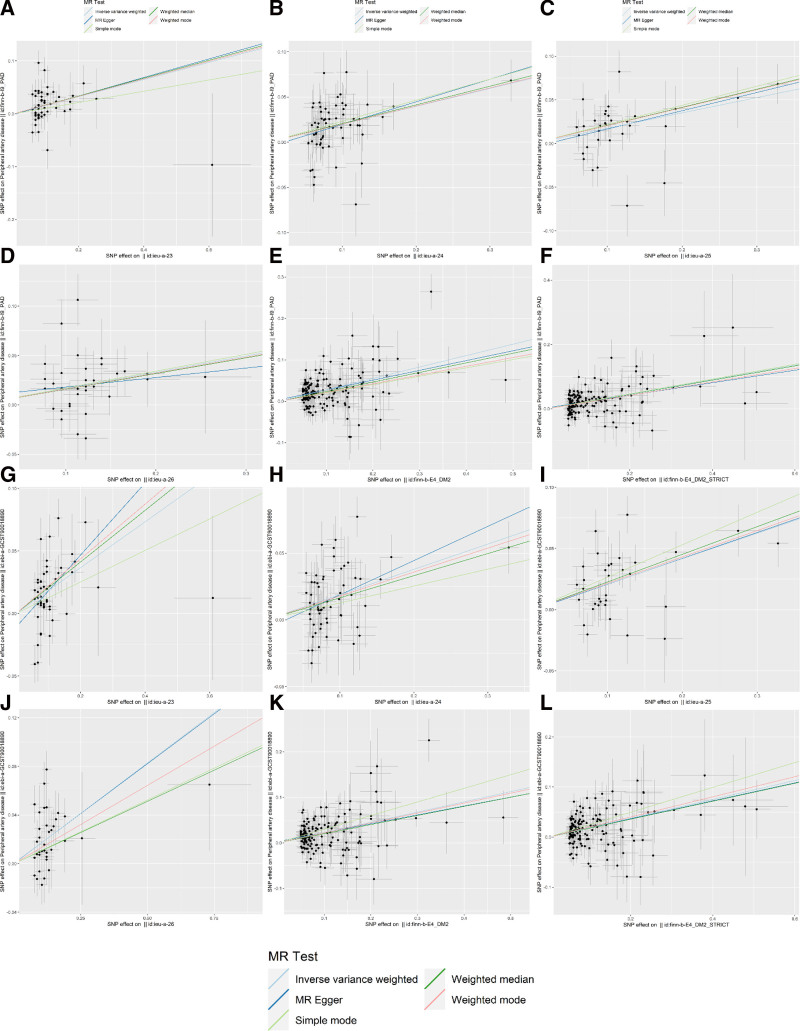
Scatterplots of MR analysis for T2D and PAD. (A) ieu-a-23 effect on finn-b-I9_PAD. (B) ieu-a-24 effect on finn-b-I9_PAD. (C) ieu-a-25 effect on finn-b-I9_PAD. (D) ieu-a-26 effect on finn-b-I9_PAD. (E) finn-b-E4_DM2 effect on finn-b-I9_PAD. (F) finn-b-E4_DM2_STRICT effect on finn-b-I9_PAD. (G) ieu-a-23 effect on ebi-a-GCST90018890. (H) ieu-a-24 effect on ebi-a-GCST90018890. (I) ieu-a-25 effect on ebi-a-GCST90018890. (J) ieu-a-26 effect on ebi-a-GCST90018890. (K) finn-b-E4_DM2 effect on ebi-a-GCST90018890. (L) finn-b-E4_DM2_STRICT effect on ebi-a-GCST90018890. MR = Mendelian randomization, PAD = peripheral artery disease, SNP = single-nucleotide polymorphisms, T2D = type 2 diabetes.

**Figure 3.**
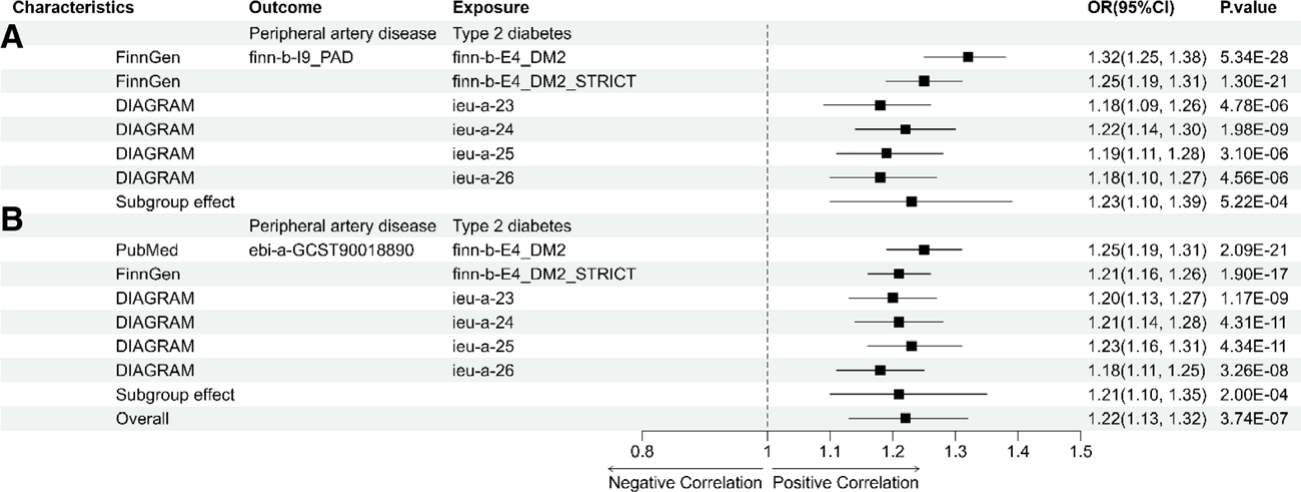
Meta-analysis of all results. (A) The outcomes are finn-b-I9_PAD, with exposures including ieu-a-23, ieu-a-24, ieu-a-25, ieu-a-26, finn-b-E4_DM2, and finn-b-E4_DM2_STRICT. (B) The outcomes are ebi-a-GCST90018890, with exposures including ieu-a-23, ieu-a-24, ieu-a-25, ieu-a-26, finn-b-E4_DM2, and finn-b-E4_DM2_STRICT. CI = confidence interval, OR = odds ratio, PAD = peripheral artery disease.

MR analyses were performed separately for the 2 datasets related to PAD. Subsequently, a meta-analysis was conducted on the results obtained from the 2 subgroups. The result that IVW (OR: 1.22, 95% CI: 1.13–1.32, *P* = 3.74e−07). The results of MR-Egger, weighted median, simple mode, and weighted mode were consistent with IVW (Fig. 2). Thus, we believe that there is a causal link between T2D and PAD. The results obtained using the leave-one-out method showed no significant sensitivity effects (Supplementary Figures S1–S12, Supplemental Digital Content, http://links.lww.com/MD/O332) and horizontal pleiotropy was not observed (*P* > .05). However, in certain subgroups, Cochran *Q* statistical test revealed sources of heterogeneity (*P* < .05) (Table 1). Despite some indications of heterogeneity, all the results consistently supported the same direction, with no evidence of horizontal pleiotropy. Moreover, the leave-one-out method enhances the robustness of the results. Integrating data from various sources has markedly minimized the influence of random errors, thereby bolstering the statistical power (Fig. 3). After obtaining the final set of SNPs (N = 376) (Supplementary Table S1, Supplemental Digital Content, http://links.lww.com/MD/O331), duplicates were removed and imported into the FUMAGWAS database for gene annotation (Supplementary Table S2, Supplemental Digital Content, http://links.lww.com/MD/O331). Subsequently, we identified these genes as potential genetic core genes associated with both T2D and PAD, totaling 126 genes (Supplementary Table S3, Supplemental Digital Content, http://links.lww.com/MD/O331).

**Table 1:** Summary of MR results.

Outcome	Exposure	Method	OR (95% CI)	*P*	*Q*_pval	Pleiotropy
finn-b-I9_PAD	ieu-a-23	IVW	1.18 (1.09–1.26)	4.78E–06	0.06	
		MR-Egger	1.19 (0.96–1.48)	1.22E–01	0.05	0.90
		Weighted median	1.18 (1.09–1.29)	1.32E–04		
		Simple mode	1.11 (1.03–1.36)	2.23E–01		
		Weighted mode	1.18 (1.03–1.36)	2.32E–02		
	ieu-a-24	IVW	1.22 (1.14–1.30)	1.98E–09	0.04	
		MR-Egger	1.28 (1.09–1.51)	4.13E–03	0.03	0.50
		Weighted median	1.23 (1.12–1.35)	1.98E–05		
		Simple mode	1.26 (1.08–1.47)	4.87E–03		
		Weighted mode	1.22 (1.10–1.36)	5.14E–04		
	ieu-a-25	IVW	1.19 (1.11–1.28)	3.10E–06	0.08	
		MR-Egger	1.23 (1.04–1.47)	2.53E–02	0.07	0.68
		Weighted median	1.23 (1.12–1.36)	3.05E–05		
		Simple mode	1.24 (1.07–1.44)	6.39E–03		
		Weighted mode	1.23 (1.11–1.36)	4.81E–04		
	ieu-a-26	IVW	1.18 (1.10–1.27)	4.56E–06	0.08	
		MR-Egger	1.10 (0.82–1.47)	5.19E–01	0.07	0.63
		Weighted median	1.17 (1.08–1.28)	2.78E–04		
		Simple mode	1.19 (1.02–1.37)	2.85E–02		
		Weighted mode	1.17 (1.04–1.33)	1.42E–02		
	finn-b-E4_DM2	IVW	1.32 (1.25–1.38)	5.34E–28	0.01	
		MR-Egger	1.27 (1.14–1.41)	2.71E–05	0.01	0.41
		Weighted median	1.26 (1.16–1.37)	1.12E–08		
		Simple mode	1.22 (1.01–1.48)	3.63E–02		
		Weighted mode	1.24 (1.11–1.37)	1.51E–04		
	finn-b-E4_DM2_STRICT	IVW	1.25 (1.20–1.31)	1.30E–21	0.1	
		MR-Egger	1.21 (1.10–1.34)	2.17E–04	0.1	0.49
		Weighted median	1.25 (1.16–1.35)	9.90E–09		
		Simple mode	1.26 (1.08–1.47)	3.93E–03		
		Weighted mode	1.23 (1.12–1.36)	6.08E–05		
ebi-a-GCST90018890	ieu-a-23	IVW	1.20 (1.13–1.27)	1.17E–09	0.003	
		MR-Egger	1.33 (1.13–1.57)	1.02E–03	0.004	0.18
		Weighted median	1.23 (1.14–1.32)	4.32E–08		
		Simple mode	1.13 (0.98–1.31)	8.92E–02		
		Weighted mode	1.24 (1.09–1.41)	1.35E–03		
	ieu-a-24	IVW	1.21 (1.14–1.28)	4.31E–11	0.003	
		MR-Egger	1.29 (1.12–1.49)	8.69E–04	0.003	0.32
		Weighted median	1.18 (1.09–1.28)	3.72E–05		
		Simple mode	1.14 (0.98–1.31)	9.04E–02		
		Weighted mode	1.20 (1.10–1.31)	1.76E–04		
	ieu-a-25	IVW	1.23 (1.16–1.31)	4.34E–11	0.02	
		MR-Egger	1.24 (1.07–1.43)	7.92E–03	0.01	0.96
		Weighted median	1.25 (1.17–1.34)	1.90E–10		
		Simple mode	1.30 (1.15–1.48)	2.15E–04		
		Weighted mode	1.24 (1.14–1.35)	1.71E–05		
	ieu-a-26	IVW	1.18 (1.11–1.25)	3.26E–08	0.011	
		MR-Egger	1.18 (1.00–1.39)	6.30E–02	0.008	0.98
		Weighted median	1.12 (1.03–1.19)	4.69E–03		
		Simple mode	1.11 (0.97–1.27)	1.35E–01		
		Weighted mode	1.14 (1.03–1.26)	1.77E–02		
	finn-b-E4_DM2	IVW	1.25 (1.20–1.31)	2.09E–21	9.19E–07	
		MR-Egger	1.22 (1.09–1.35)	4.85E–04	7.99E–07	0.55
		Weighted median	1.22 (1.14–1.31)	8.49E–09		
		Simple mode	1.35 (1.15–1.58)	2.92E–04		
		Weighted mode	1.24 (1.13–1.37)	9.67E–06		
	finn-b-E4_DM2_STRICT	IVW	1.21(1.16, 1.26)	1.90E–17	9.97E–05	
		MR-Egger	1.20 (1.08–1.32)	4.91E–04	8.14E–05	0.84
		Weighted median	1.20 (1.16–1.28)	4.46E–07		
		Simple mode	1.28 (1.11–1.48)	1.19E–03		
		Weighted mode	1.22 (1.12–1.34)	2.27E−05		

CI = confidence interval, IVW = inverse variance weighting, MR = Mendelian randomization, OR = odds ratio, PAD = peripheral artery disease, pleiotropy = *P* value of the pleiotropy test, *Q*_pval = *P* value of the Cochrane *Q* statistical test.

### 3.2. Results of the DEGs

**Figure 4.**
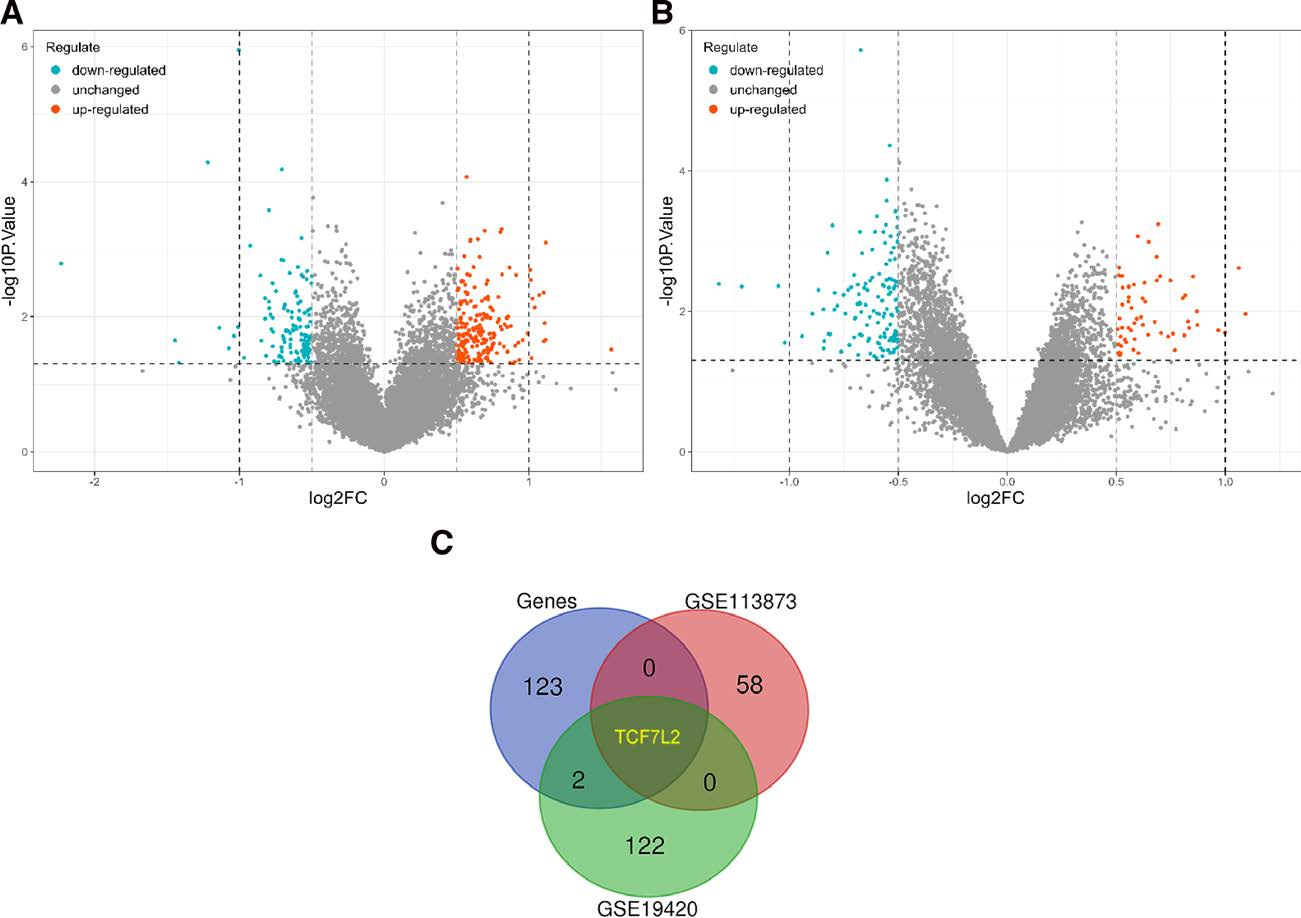
Results of differential gene analysis. (A) Differential gene expression in the GSE19420 (T2D). (B) Differential gene expression in the GSE113873 (PAD). (C) The intersection results of gene sets related to genetic effects, the differential gene set GSE19420, and the differential gene set GSE113873 are acquired. FC = fold change, PAD = peripheral artery disease, T2D = type 2 diabetes.

We performed analyses of differential gene expression on the GSE19420 (T2D) and GSE113873 (PAD) datasets, respectively. In the first dataset, 23,322 genes were identified, of which 209 were upregulated and 125 were downregulated (Fig. 4A). In the second dataset, 19,916 genes were identified, of which 59 were upregulated and 129 were downregulated (Fig. 4B).

### 3.3. Results of the WGCNA

**Figure 5.**
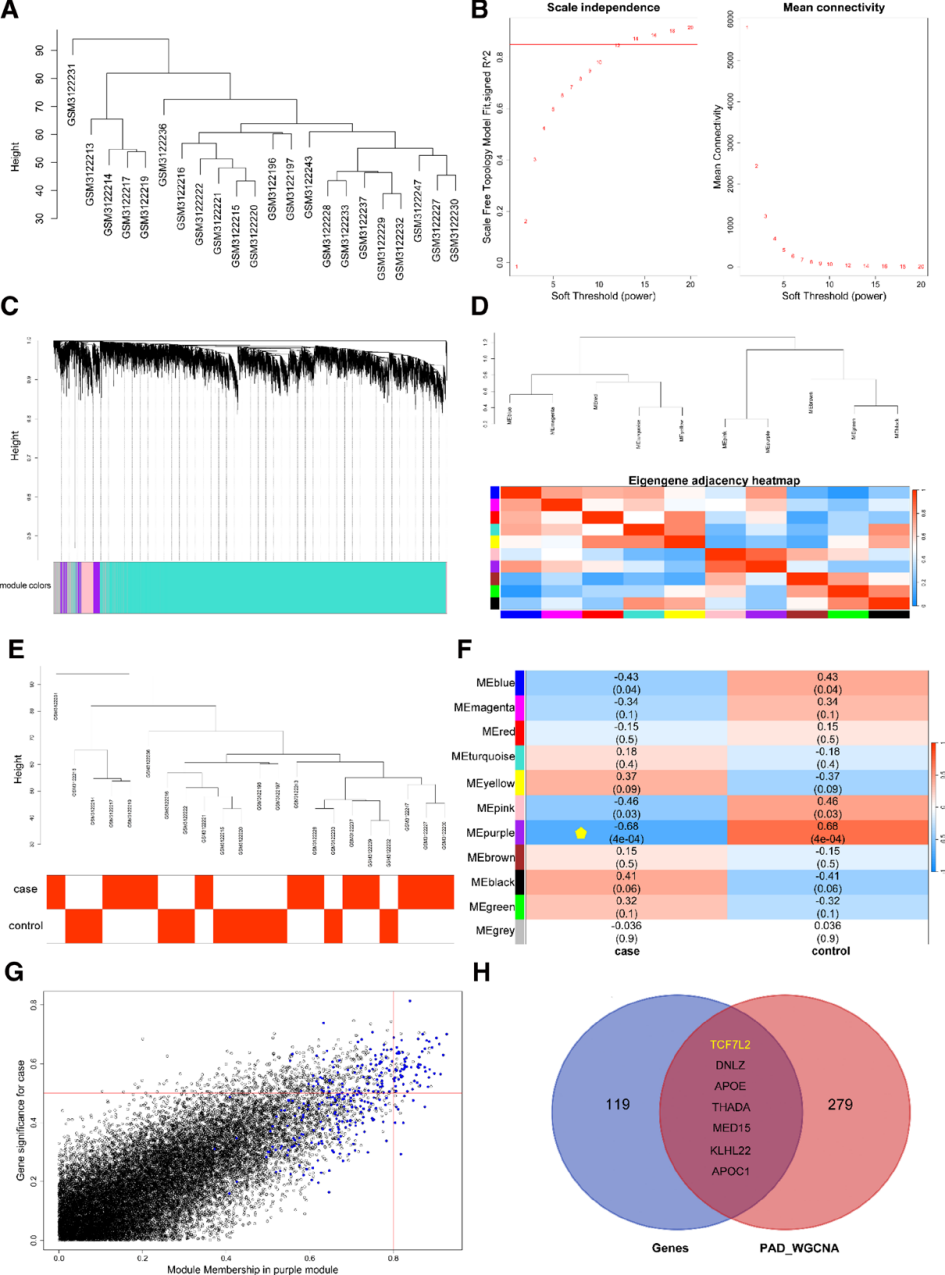
Results of WGCNA analysis. (A) Sample clustering to detect outliers. (B) Soft threshold (power). (C) Cluster dendrogram. (D) Eigengene adjacency heatmap. (E) Sample dendrogram and trait heatmap. (F) Module–trait relationships. (G) Scatter plot of module membership versus gene significance (all genes). (H) The intersection results of gene sets related to genetic effects and the purple module gene set. PAD = peripheral artery disease, WGCNA = weighted gene coexpression network analysis.

The gene expression matrix GSE113873 was subjected to WGCNA. After excluding unsuitable patients, 22 clinical samples were included in the study (Fig. 5A). Using the presented data, we set a soft threshold of 12 (*R*^2^ = 0.85) to construct a scale-free network (Fig. 5B). Pearson correlation coefficients were employed to cluster the samples in the expression matrix network (Fig. 5C). Subsequently, we constructed an adjacency matrix and created a TOM (Fig. 5D, E). Using mean hierarchical clustering and dynamic tree partitioning, 11 discrete modules were identified (Fig. 5F). A strong correlation between the purple module and the clinical phenotypes was observed. Consequently, we chose to further analyze this module because of its clinical significance. We calculated the correlation of each gene with both modules and phenotypic traits and identified significant associations between the genes in the purple module and clinical phenotypes (Fig. 5G). Finally, we extracted the genes from the purple module for further investigation.

### 3.4. Results of overall T2D to PAD

We detected a substantial increase in the risk of PAD associated with T2D using MR analysis. The results of the extensive meta-analysis provide additional support and reinforce the aforementioned conclusions. Subsequently, we identified genetic gene sets that were closely associated with both exposure and outcome. This gene set was subsequently compared with DEGs in GSE19420 (T2D) and GSE113873 (PAD). Our findings suggest that *TCF7L2* was a shared gene that was upregulated in GSE19420 (T2D) and downregulated in GSE113873 (PAD) (Fig. 4C). In the WGCNA of GSE113873 (PAD), we noted a robust correlation between the purple module and clinical phenotypes, notably manifested by a downregulation trend. We isolated the internal genes within the purple module and compared them to the gene sets strongly associated with both exposure and outcome (Fig. 5H). These findings suggest that *TCF7L2* is a gene shared between the 2 conditions. Based on these findings, it is clear that T2D markedly increases the risk of PAD. Concurrently, *TCF7L2* has been implicated in crucial pathogenic mechanisms under both conditions.

## 4. Discussion

In the initial phase, we observed a direct and positive association between T2D and PAD. After excluding the influence of horizontal pleiotropy, all outcomes consistently converged, ensuring the stability of the MR results despite certain heterogeneity effects. We performed a meta-analysis of all outcomes. The findings from the individual subgroups and the overall summary aligned with the MR results bolstered the reliability of our conclusions. This conclusion is consistent with the long-term clinical observations.

The augmentation of blood glucose concentration precipitates the production of advanced glycation end products (AGEs).^[42]^ AGEs intensify endothelial cell glycotoxicity, resulting in a pronounced elevation in oxidative and inflammatory reactions within the vascular endothelium,^[43]^ which manifests as impaired vascular endothelial function in the early stages of the disease.^[44]^ Normal endothelial structure and function are fundamental to vascular health and are closely associated with vascular diseases and mortality.^[45]^ Chronic inflammation can damage endothelial cells, compromising vascular endothelium integrity, leading to platelet aggregation,^[46]^ red blood cell clumping, fibrinogen accumulation, and formation of blood clots.^[44]^ Venous thromboembolism poses a serious threat to human health,^[47]^ making the prevention of thrombosis particularly important.^[48,49]^ A close connection between high blood sugar and innate immune cell inflammation has been established through the modification of cellular functions,^[50]^ release of inflammatory factors, and exacerbation of the inflammatory response.^[51]^ In addition, the interaction between AGEs and low-density lipoprotein can induce the generation of proinflammatory factors, affecting macrophage phagocytic activity and smooth muscle cell phenotypic transformation.^[52]^ In adipose tissue, macrophages polarize into the proinflammatory M1 type, secreting numerous proinflammatory cytokines that damage insulin signaling,^[53]^ promoting insulin resistance and inflammatory responses.^[54]^ Effective management of elevated blood glucose levels is essential,^[55]^ involving the simultaneous suppression of inflammatory and oxidative stress responses to delay the onset of diabetes and prevent vascular complications.^[56]^

Reactive oxygen species (ROS) production is a pivotal factor contributing to vascular damage in hyperglycemia-induced diabetic complications.^[57]^ Elevated blood glucose levels significantly increase ROS production by activating the protein kinase C pathway,^[58]^ enhancing the hexosamine pathway, and augmenting AGE generation in subsequent stages.^[59]^ During the oxidative stress response, excessive ROS accumulation stimulates oxidized-low-density lipoprotein and AGE generation, exacerbating vascular damage,^[60]^ inhibiting endothelial nitric oxide synthase activity, and inducing endothelial cell dysfunction.^[61]^ Elevated ROS levels contribute to inflammation, endothelial dysfunction, plaque instability, and atherosclerosis.^[62]^ Elevated blood sugar impedes DNA self-repair mediated by ROS, thereby worsening atherosclerosis.^[63]^ The impact of T2D on lipid metabolism is now recognized as a risk factor for atherosclerosis development.^[64]^ Addressing dyslipidemia in diabetes helps lower the risk of vascular complications.^[65]^ Diabetes is frequently associated with endothelial dysfunction, resulting in decreased bioavailability of NO.^[66]^ This inhibition impedes microvessel dilation, contributing to microcirculation disorders.^[67]^ The research reports that T2D imposes a significant burden on the autonomic and somatic nervous systems.^[68,69]^ The vascular and nervous systems share close connections with common molecular mechanisms.^[70]^ Whether diabetes increases the risk of vascular disease by affecting the nervous system requires robust evidence. In patients with T2D, the risk of PAD significantly increases owing to multiple contributing factors, with sustained hyperglycemia being a primary pathological factor.

In the second phase of the study, we synthesized findings from the GEO data analysis, revealing the pivotal role of *TCF7L2* in the pathogenesis of both T2D and PAD. The expression patterns of *TCF7L2* exhibited distinct trends in the T2D and PAD groups, indicating its potential involvement in distinct biological processes for each condition. Nevertheless, our confidence in the close association between *TCF7L2* and the onset of both conditions remains unwavering.

The rs7903146 variant within *TCF7L2* is acknowledged as the most crucial allele associated with diabetes.^[71]^ Limited studies have suggested that genetic variations in *TCF7L2* rs7903146 might elevate the risk of peripheral arterial disease in patients with long-term T2D (OR: 2.595, 95% CI: 1.177–5.722).^[72]^ The reduction in TCF7L2 protein expression was associated with decreased levels of glucagon-like peptide 1 and glucose-dependent insulinotropic polypeptide, which led to the inhibition of insulin secretion induced by GLP-1 and GIP.^[73]^ This downregulation subsequently contributes to the impaired functionality of pancreatic β cells. The report suggested that downregulation of *TCF7L2* gene expression might induce apoptosis and impair the function of pancreatic β cells.^[74]^ Conversely, increasing TCF7L2 expression is considered crucial for protecting pancreatic islets against apoptosis and functional impairment induced by high glucose and cytokines.^[75]^ This has highlighted the significance of upregulating TCF7L2 to safeguard pancreatic islets from apoptosis and functional deterioration caused by exposure to elevated glucose levels and cytokine induction.

In summary, *TCF7L2* rs7903146 is a high-risk gene for T2D. However, it also plays a significant role in modulating the endothelial inflammation and oxidative stress responses. The regulatory effect leads to dysfunction of endothelial cells, damage to pancreatic β cells, disruption of lipid metabolism, dysfunction of the autonomic nervous system, atherosclerosis, and injury to vascular tissues. We speculate that the elevated blood glucose level in T2D is the primary exacerbating factor for PAD.

This study had certain limitations. The initial part of our study integrated datasets from multiple cohorts. While applying the standard criteria for IVs (*P* = 5e−08), a few individual datasets did not yield statistically significant IVs representing the relationship of interest. Consequently, we adopted a more flexible screening criterion (*P* = 5e−06) to consider the relevant datasets inclusively. A degree of heterogeneity was observed in the ebi-a-GCST90018890 analysis. No distinct sources of sensitivity SNPs were identified using the leave-one-out method. To mitigate the risk of population stratification, we assessed only individuals of European ancestry and excluded other ancestral groups. In the second phase of our study, constrained by available data in GEO, our analysis exclusively concentrated on scrutinizing expression matrices derived from skeletal muscle, lacking data from alternative sources. During the analysis of differential gene expression, we adjusted the criteria for upregulating and downregulating genes to a more flexible range (log FC: 0.5 and −0.5), aiming to enhance the identification of a broader spectrum of DEGs.

## 5. Conclusions

In this study, we found that T2D significantly increased PAD risk. We also predicted a robust correlation between *TCF7L2* and its functional protein expression in patients with T2D and PAD. *TCF7L2* warrants further investigation.

## Acknowledgments

The authors appreciate the comprehensive GWAS and GEO public data compilations and extend their thanks to all participants in these studies. Special acknowledgments was given to the developers and contributors of the R programming language.

## Author contributions

**Conceptualization:** Jie Liu.

**Writing – original draft:** Jie Liu.

**Methodology:** XingDe Liu.

**Formal analysis:** Rui Rao.

**Writing – review & editing:** Wen Li.

## Supplementary Material


