# Supplementary material for: TCF7L2 as a target of peripheral artery disease in patients with type 2 diabetes: A 2-sample Mendelian randomization and bioinformatics study
Source: Medicine (Baltimore). 2025 Feb 14;104(7):e41431. doi: 10.1097/MD.0000000000041431 (PMC11835089; doi:10.1097/MD.0000000000041431)
Supplement: Supplementary file 2 [file medi-104-e41431-s002.pdf]

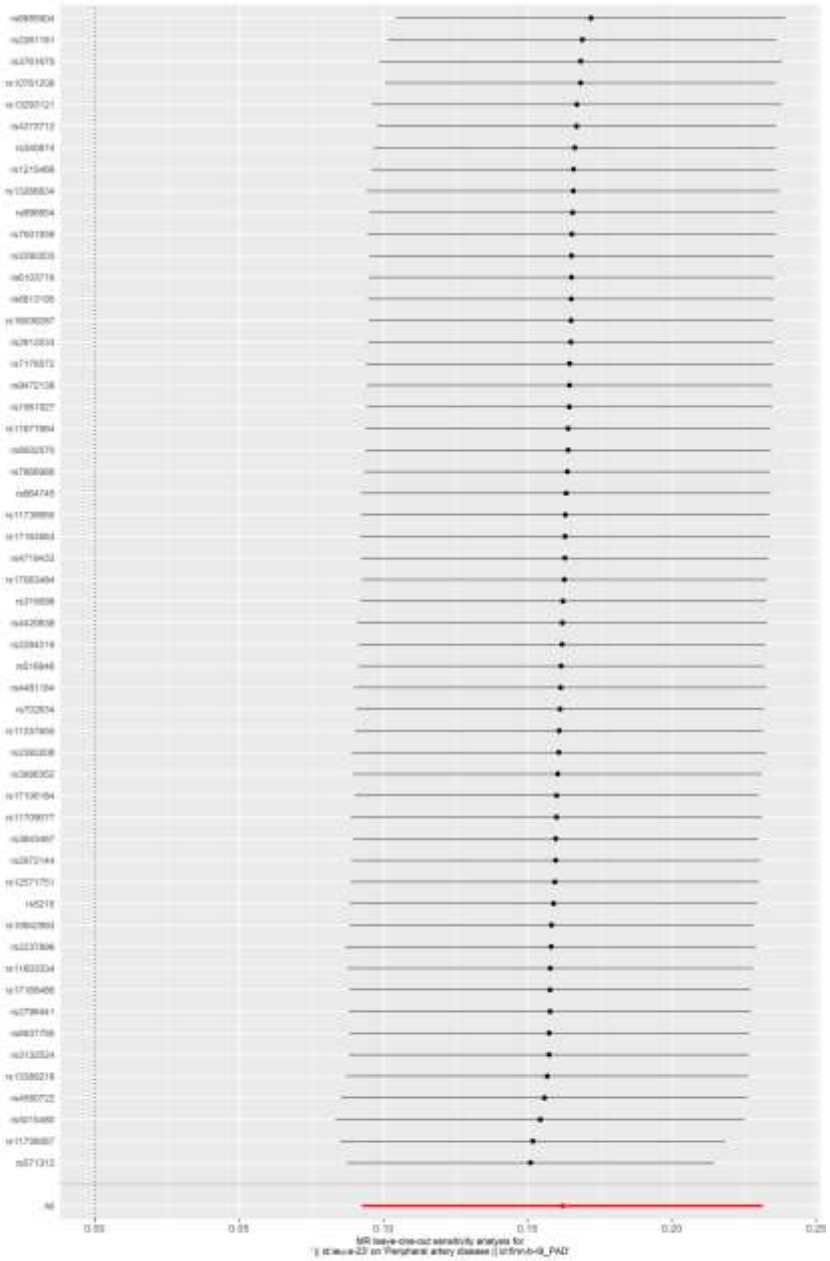

Supplementary Figure S1

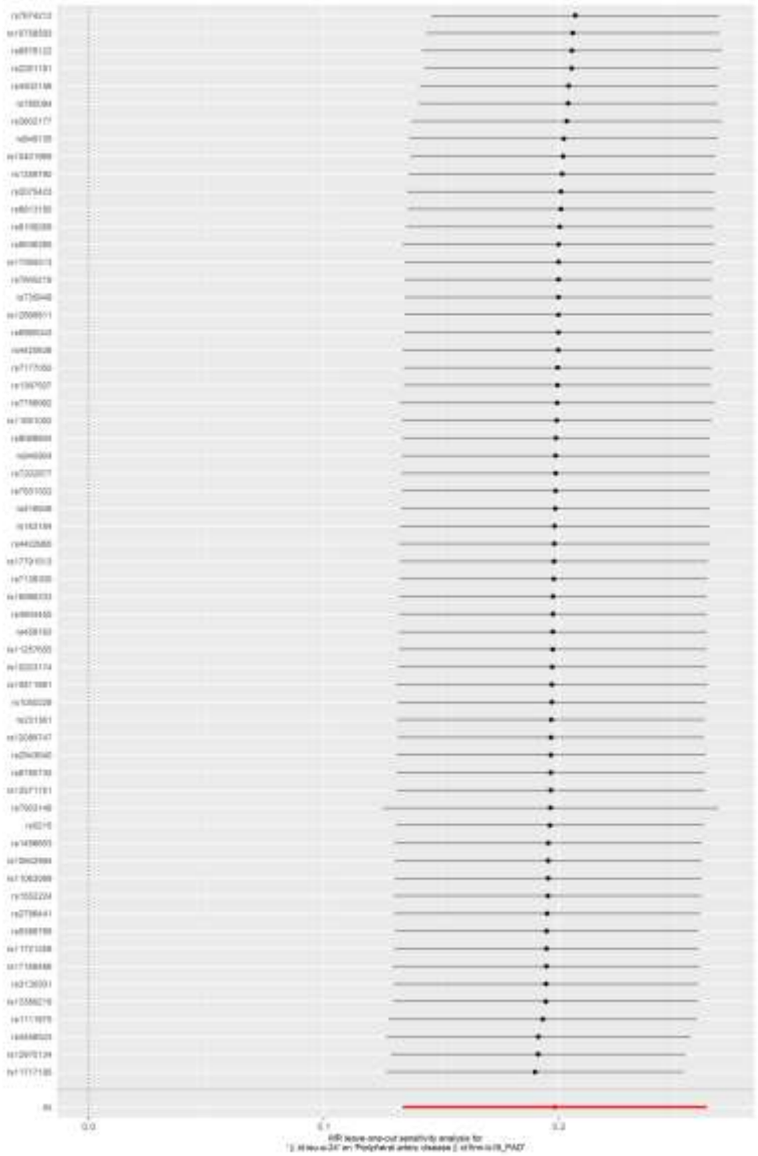


Supplementary Figure S2

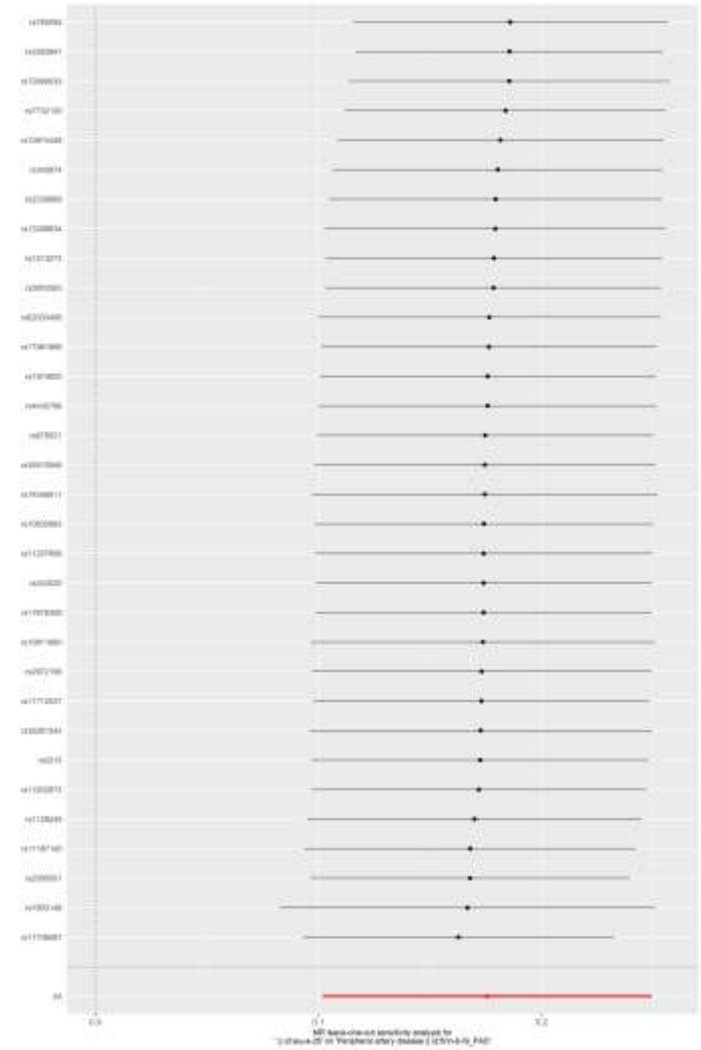


Supplementary Figure S3

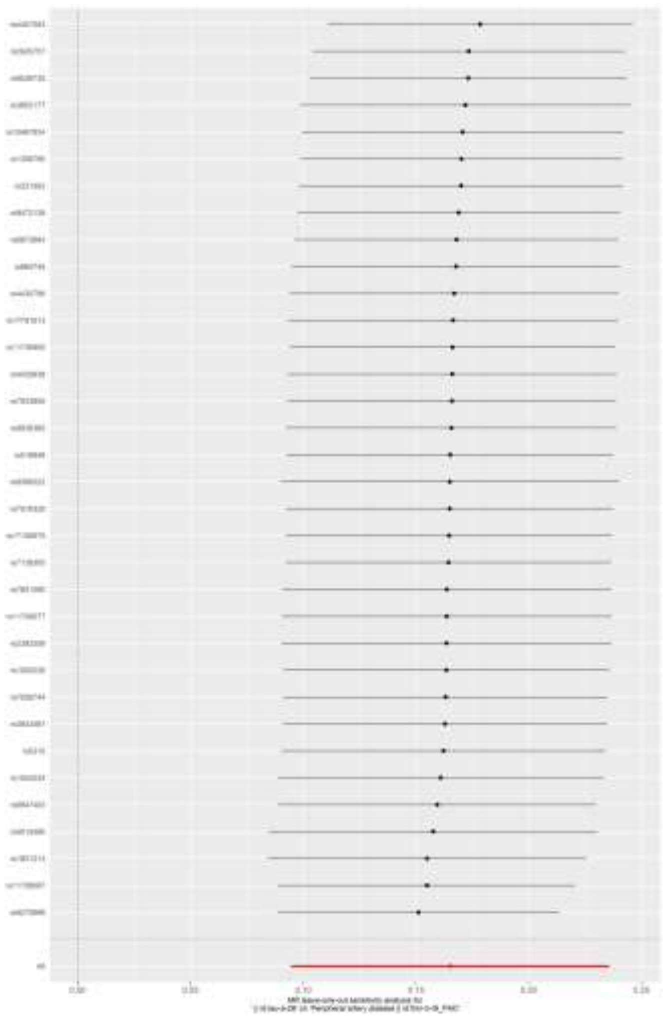

Supplementary Figure S4

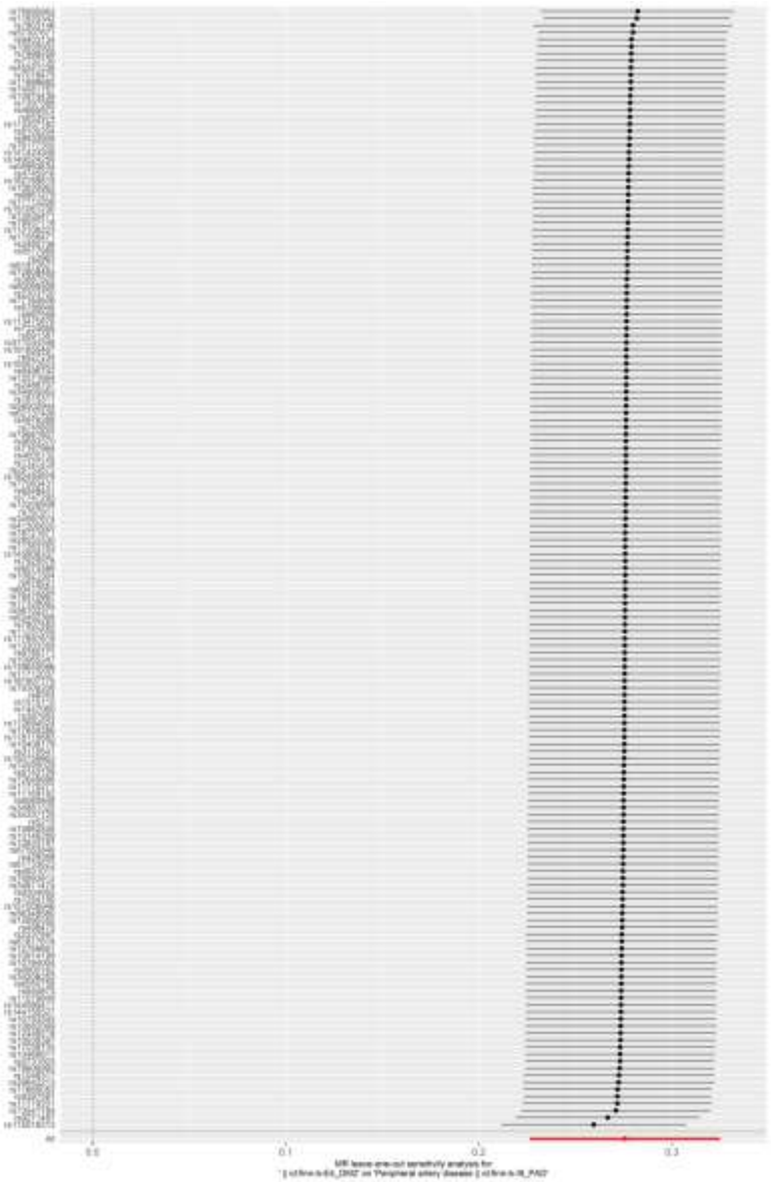


Supplementary Figure S5

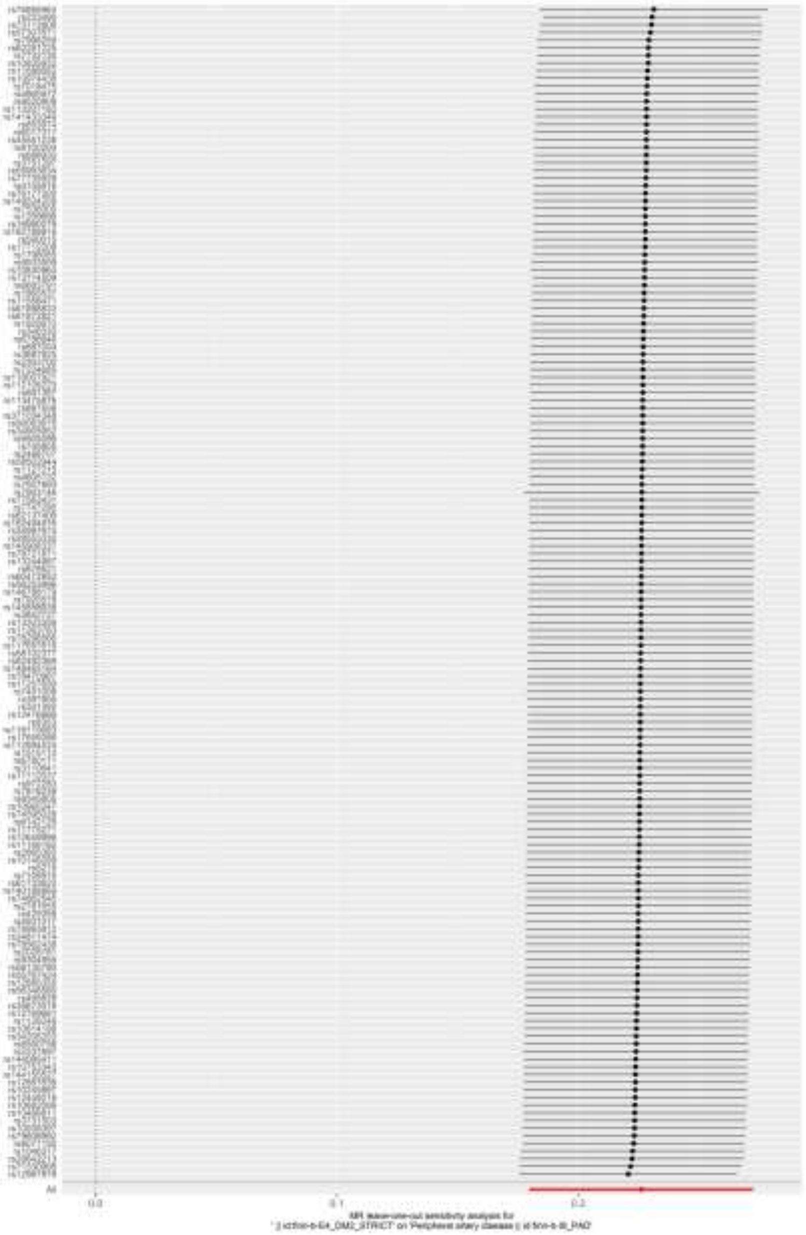


Supplementary Figure S6

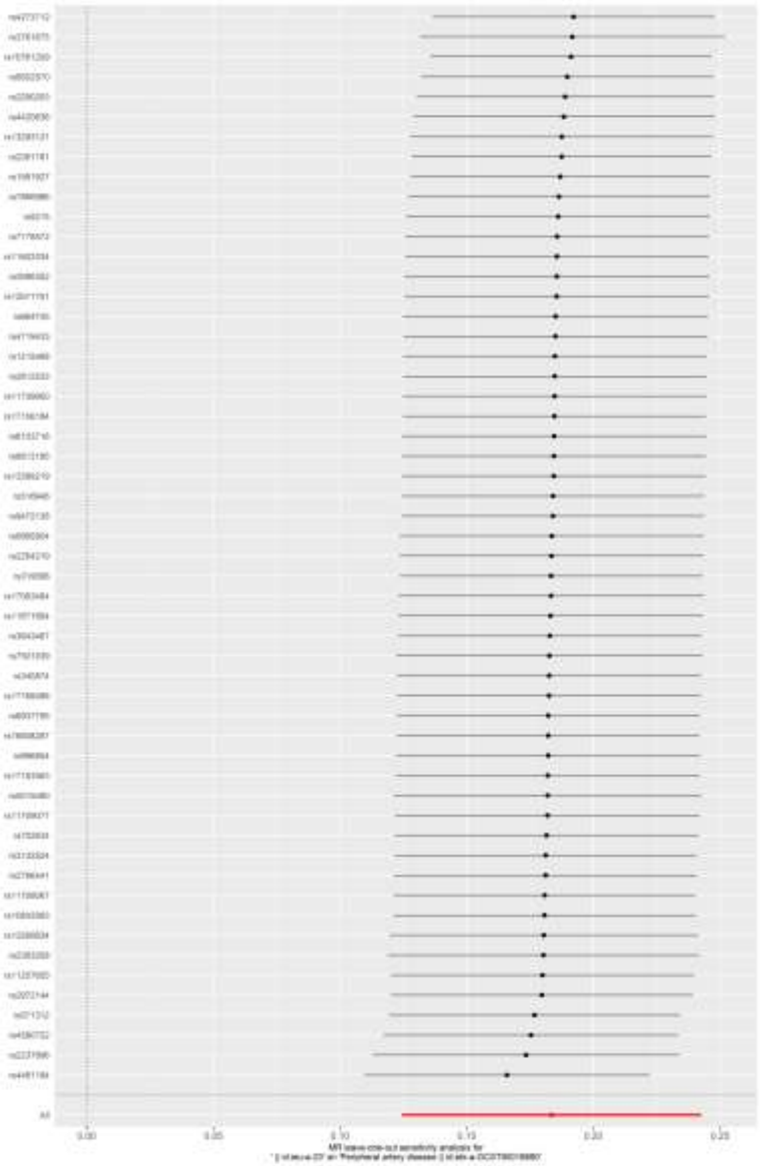


Supplementary Figure S7

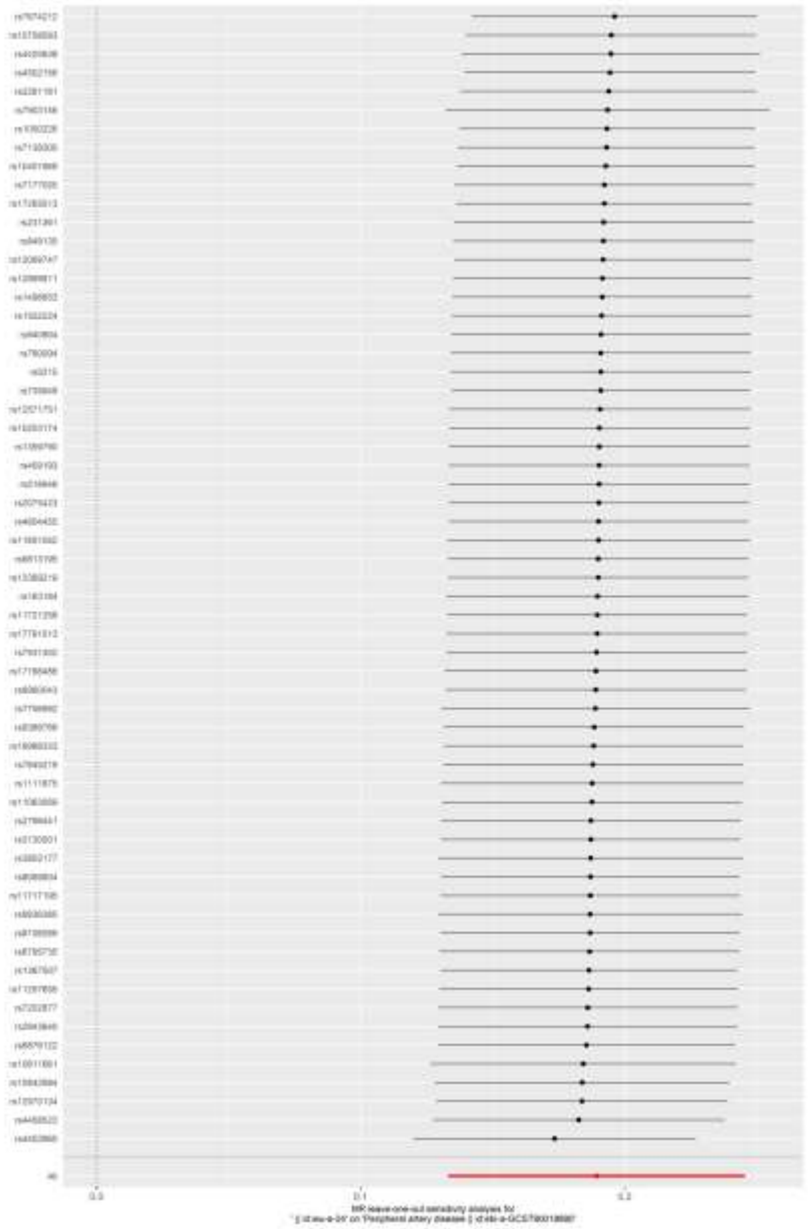

Supplementary Figure S8

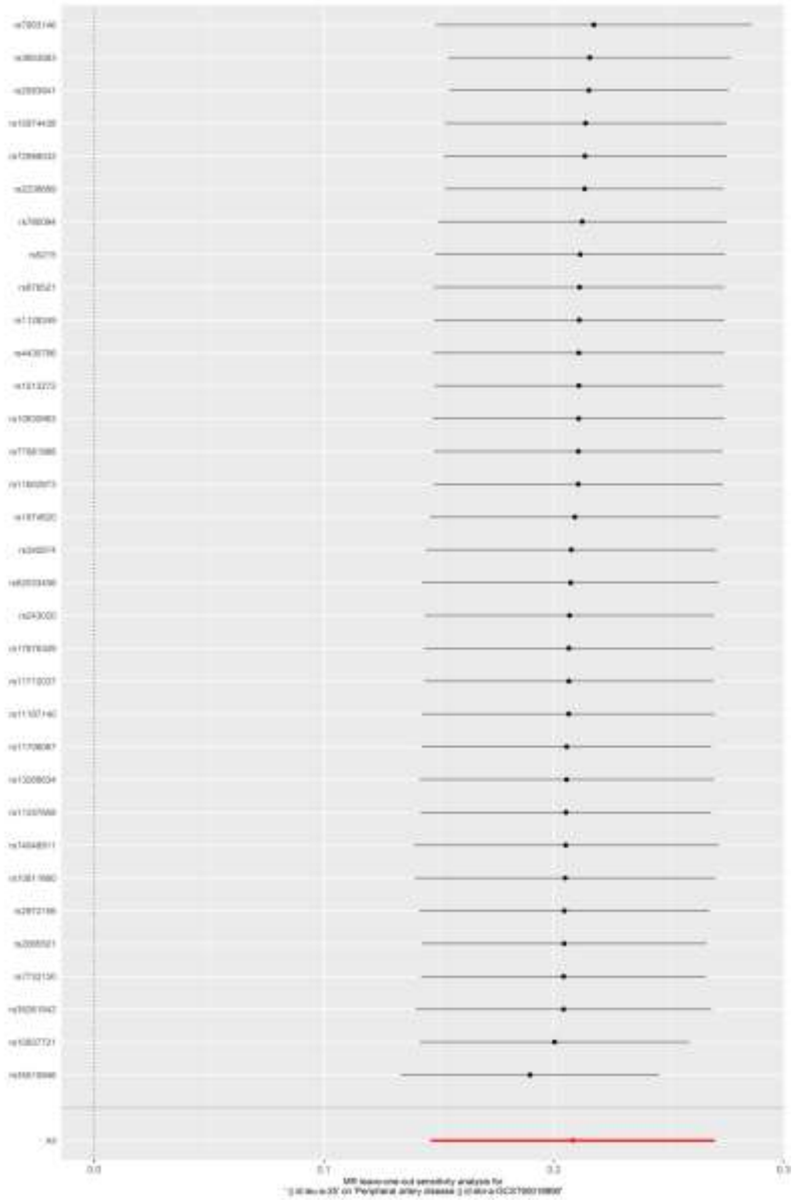

Supplementary Figure S9

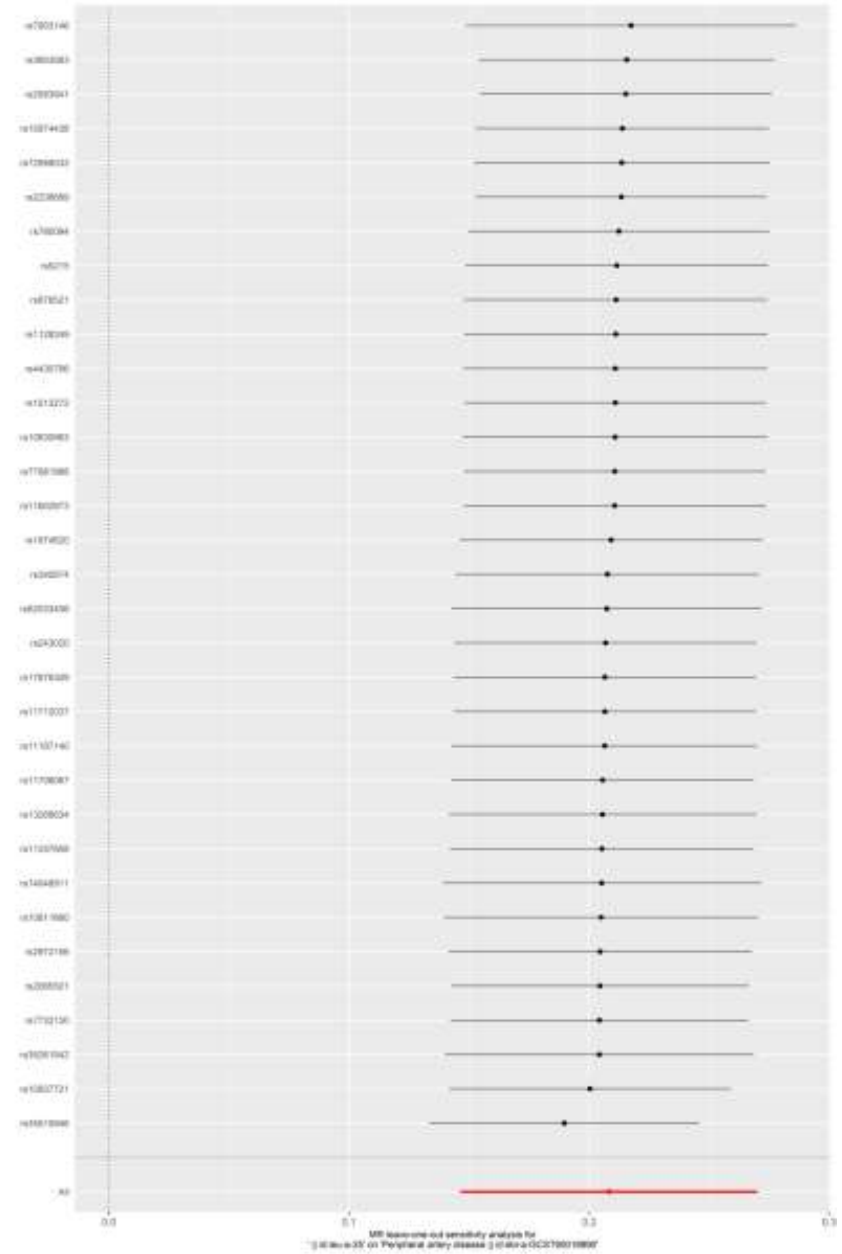

Supplementary Figure S10

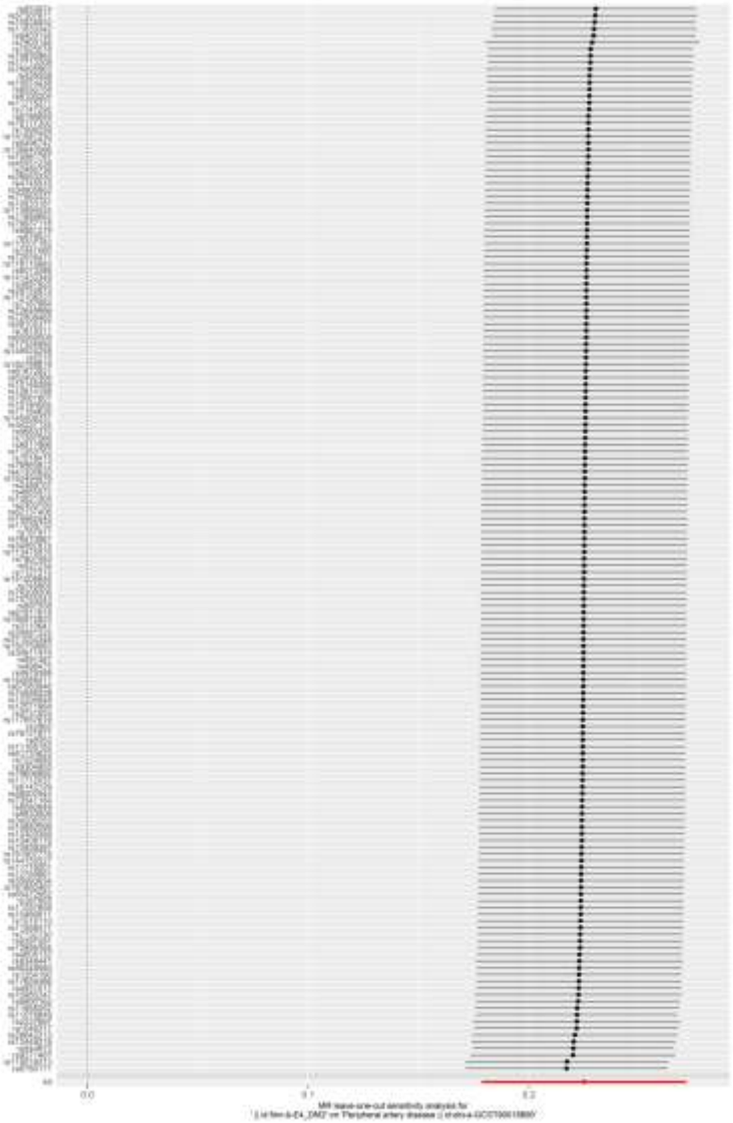


Supplementary Figure S11

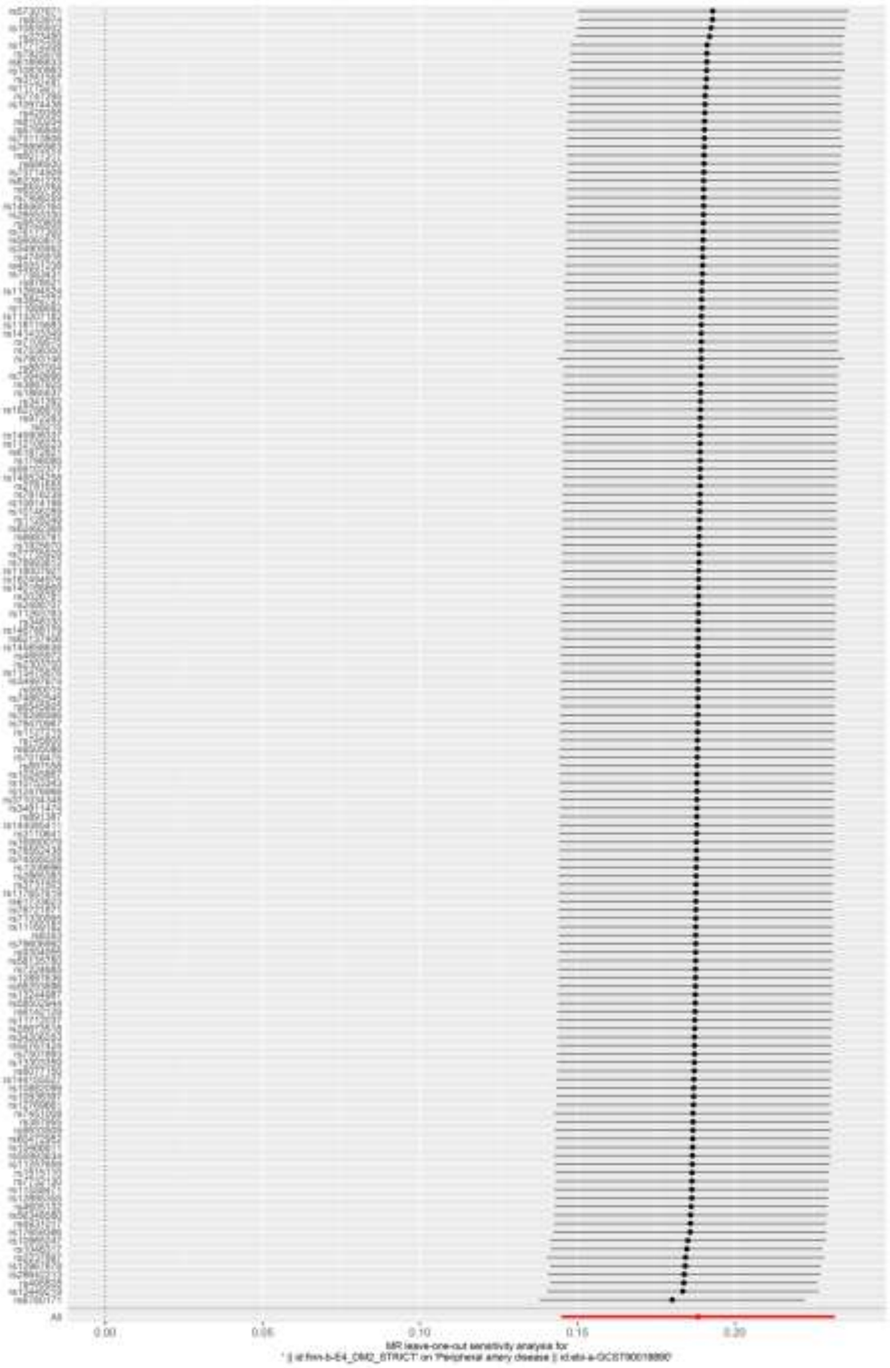


Supplementary Figure S12
